# Deletion of *alpB* Gene Influences Outer Membrane Vesicles Biogenesis of *Lysobacter* sp. XL1

**DOI:** 10.3389/fmicb.2021.715802

**Published:** 2021-08-16

**Authors:** Irina V. Kudryakova, Alexey S. Afoshin, Tanya V. Ivashina, Natalia E. Suzina, Elena A. Leontyevskaya, Natalia V. Leontyevskaya (Vasilyeva)

**Affiliations:** ^1^Laboratory of Microbial Cell Surface Biochemistry, Pushchino Center for Biological Research, G. K. Skryabin Institute of Biochemistry and Physiology of Microorganisms, Russian Academy of Sciences, Pushchino, Russia; ^2^Laboratory of Molecular Microbiology, Pushchino Center for Biological Research, G. K. Skryabin Institute of Biochemistry and Physiology of Microorganisms, Russian Academy of Sciences, Pushchino, Russia; ^3^Laboratory of Microbial Cytology, Pushchino Center for Biological Research, G. K. Skryabin Institute of Biochemistry and Physiology of Microorganisms, Russian Academy of Sciences, Pushchino, Russia

**Keywords:** biogenesis of vesicles, *Lysobacter* sp. XL1, antimicrobial potency of vesicles, deletion in gene *alpB*, bacteriolytic protease L5

## Abstract

Outer membrane vesicles (OMVs) produced by Gram-negative bacteria constitute important factors in defining interactions with the extracellular milieu. *Lysobacter* sp. XL1 produces OMVs capable of lysing microbial cells due to the presence in their cargo of bacteriolytic protease L5 (AlpB). Although protein L5 has been functionally and biochemically characterized (including aspects of its packing into OMVs), its role in vesicle biogenesis through genetic deletion of *alpB* had not been studied previously. Here, we have successfully deleted *alpB* by allelic replacement and show that the *alpB* deletion mutant produces a significantly lower amount of OMVs that lack bacteriolytic activity and display altered ultrastructural characteristics in relation to the OMVs produced by the wild-type strain. These results confirm that, as previously proposed, protein L5 participates in OMV production through a mechanism that is not yet fully understood.

## Introduction

Outer membrane vesicles (OMVs) are structures 50–300 nm in diameter that all Gram-negative bacteria form from their outer membrane (OM). OMVs perform a number of vital activities in relation to nutrient uptake, microbe–microbe interactions (including horizontal gene transfer), pathogen–host interactions, symbiotic interactions, and cell protection like phage and toxin release (Mashburn-Warren and Whiteley, 2006; Avila-Calderón et al., 2015; Schwechheimer and Kuehn, 2015; Guerrero-Mandujano et al., 2017; Nagakubo et al., 2020). In light of this, the topicality of vesicle studies is evident.

At present, one of the most intriguing issues in vesicle subject matter is OMV biogenesis. At first, vesicles were considered to form due to spontaneous evagination of the OM, followed by the release of these fragments (Knox et al., 1966). Later on, three models of vesicle biogenesis were formulated. According to the first model, vesicles form in the region of a decreased concentration of lipoproteins (Hoekstra et al., 1976; Deatherage et al., 2009; Schwechheimer et al., 2014). The second model explains the presence of cell debris [misfolded proteins, peptidoglycan and lipopolysaccharide (LPS) fragments] in vesicles (Zhou et al., 1998; Hayashi et al., 2002; Schwechheimer et al., 2014). According to the third model, the formation of vesicles occurs under the action of curvature-inducing molecules (B-type LPS, signalling molecule 2-heptyl-3-hydroxy-4-quinolone (PQS) (Kadurugamuwa and Beveridge, 1995; Mashburn-Warren and Whiteley, 2006; Schertzer and Whiteley, 2012). Thus, all these models indicate that vesicles form from OM destabilization loci, and their contents are periplasmic components randomly captured in the process of vesicle formation.

With time, the view of vesicle biogenesis began to change. Data supporting a model for the specific sorting of vesicle components emerged (Horstman and Kuehn, 2000; Kato et al., 2002; Haurat et al., 2011, 2015; Schwechheimer and Kuehn, 2015). This contributed to the emergence of the theory of the formation of various vesicle groups (subpopulations) produced by one cell type (Olofsson et al., 2010; Rompikuntal et al., 2012; Kunsmann et al., 2015; Kudryakova et al., 2015; Afoshin et al., 2020). It should be noted that this theory enables understanding the ability of vesicles to perform various vital activities of the bacterial cell. It is evident that a relation between vesicle biogenesis processes and component sorting is bound to exist. It is hypothesized that these components themselves are factors contributing to vesicle biogenesis. We addressed this hypothesis by studying of vesicle biogenesis in *Lysobacter* sp. XL1.

The Gram-negative bacterium *Lysobacter* sp. XL1 has been studied at our laboratory for about 45 years. This bacterium is a potent producer of a complex of extracellular bacteriolytic enzymes. To date, five bacteriolytic enzymes (L1–L5) have been isolated and partially characterized. By the specificity of their action on bacterial peptidoglycans, the lytic enzymes of *Lysobacter* sp. XL1 are endopeptidases (L1, L4, L5), amidase (L2), and a muramidase (L3). The bacteriolytic complex is highly efficient in breaking down competitive cells of bacteria, yeasts, mycelial fungi and some protozoa (Kulaev et al., 2006). This property makes *Lysobacter* sp. XL1 an active participant of microbial biocenoses in nature and a biotechnologically promising strain producing novel antimicrobial agents. In 2008, we found *Lysobacter* sp. XL1 to form vesicles that lysed cells of Gram-positive bacteria, yeasts, mycelial fungi and possessed a curative action with respect to staphylococcal and anthrax infections. The lytic action of vesicles is determined by bacteriolytic enzyme L5, which is a component of their cargo (Vasilyeva et al., 2008, 2014). Research into the OMV biogenesis of *Lysobacter* sp. XL1 found their formation to occur in OM segments enriched with cardiolipin (Kudryakova et al., 2017). Fractionation of vesicles revealed a group/subpopulation of vesicles containing protein L5. Electron immunocytochemistry of *Lysobacter* sp. XL1 cell sections established protein L5 to concentrate in certain loci of the periplasm at the inner leaflet of the OM; it is from these loci that vesicles form (Kudryakova et al., 2015). We hypothesized that protein L5 could participate in vesicle biogenesis. To study this, the gene of protein L5 (*alpB*) was expressed in cells of a phylogenetically close genus *Pseudomonas fluorescens* Q2-87/B. It was found that in the recombinant strain, protein L5 was contained in vesicles. Cells of the recombinant strain were shown to form a larger number of vesicles as compared with the parent strain (Kudryakova et al., 2017). All these data indirectly confirmed the involvement of bacteriolytic protein L5 in the biogenesis of that group of vesicles by means of which it was released into the extracellular milieu. To substantiate this model, the goal of the present study to investigate OMV production by an *alpB* mutant strain compared with that of the wild-type strain. We have deleted *alpB* in *Lysobacter* sp. XL1, which in itself is an important experimental accomplishment mainly because the ill-developed molecular tools available for conducting genetic studies on *Lysobacter* had not previously permitted gene knockouts. Here, we report that the quantity of vesicles produced by the mutant strain was significantly lower than that of the wild-type strain and the lytic properties of mutant strain vesicles were lost practically completely, confirming that protein L5 influences OMV formation.

## Materials and Methods

### Bacterial Strains, Plasmids, and Growth Conditions

All bacterial strains and plasmids used are listed in Table 1.

**TABLE 1:** Strains and plasmid used in this study.

Strains and plasmids	Characteristics	References
*Lysobacter* sp. XL1	Wild-type	Kulaev et al., 2006
*Lysobacter* sp. XL1Δ*alpB::tet*	Deletion in *alpB* gene (mutant form)	This study
*Lysobacter* sp. XL1Δ*alpB::alpB*	Complementation of mutant strain (complemented form)	This study
*S. aureus* 209P	Wild-type	*
*M. roseus* B-1236^T^	Wild-type	VKM B-1236
*M. luteus* B-1813^T^	Wild-type	VKM B-1813
*B. cereus* B-454^T^	Wild-type	VKM B-454
*E. coli* XL1-Blue	*recA1 endA1 gyrA96 thi hsdR17 supE44 relA1 lac/[*F′:Tn10 *proAB* + *lacI^*q*^ lacZ*Δ*M15 traD36]*	Bullock et al., 1987
pJQ200SK	Suicide vector with a *sacB* gene, Gm^*R*^	Quandt and Hynes, 1993
pBR322	Source of Tc^R^ cassette	Balbás et al., 1986
pJQ200SKΔ*5*′*alpB*	pJQ200SK with 924-bp DNA fragment containing 3′ end of *alpB* and downstream region	This study
pJQ200SKΔ*alpB*	pJQ200SK with 880-bp deletion in *alpB*	This study
pJQ200SKΔ*alpB::tet*	pJQ200SK carrying deletion in *alpB* marked with 1.43-kb Tc^R^ cassette	This study
pJQ200SKΔ*alpB::alpB*	pJQ200SK carrying the full-length *alp*B with downstream and upstream genome sequences	This study

*Kindly provided by Federal Budget Institution of Science «State Research Center for Applied Microbiology and Biotechnology».

*Escherichia coli* strain XL1-Blue used for molecular cloning was grown in a Luria–Bertani (LB) medium containing (g/l): tryptone, 10; yeast extract, 5; NaCl, 10; pH 7.0 at 37°C. Wild-type *Lysobacter* sp. XL1, *Lysobacter* sp. XL1Δ*alpB*::*tet*, and *Lysobacter* sp. XL1Δ*alpB::alpB* strains were grown at 29°C in modified LB (LB-M) broth (in g/l: peptone, 5; yeast extract, 5; NaCl, 5; pH 7.5). *Lysobacter* cultures were grown for 20 h, which corresponds to the end of the exponential growth phase. Antibiotics were added as necessary at the following final concentrations (μg/ml): for *E. coli*: tetracycline (Tc), 10; gentamicin (Gm), 10; ampicillin (Ap), 100; for *Lysobacter* sp. XL1: Tc, 10; Gm, 20. For screening clones that had undergone double crossover events, sucrose was added to a final concentration of 10%. When plates of solid media were required, agar at a concentration of 15 g/L was added to the corresponding broths.

Bacterial cultures used in lysis tests (*Staphylococcus aureus* 209P, *Micrococcus luteus* B-1813, *Micrococcus roseus* B-1236, and *Bacillus cereus* B-454) were grown at 29°C as confluent lawns on plates of IBPM RAS medium (in g/l: yeast extract, 1; soybean extract, 30; tryptone, 5; aminopeptide, 60; agar, 15; pH 7.2).

### DNA Manipulations

DNA recombinant techniques were performed according to standard procedures (Sambrook and Russell, 2001). All restriction endonucleases, T4 polynucleotide kinase, shrimp alkaline phosphatase, and T4 DNA ligase were obtained from Thermo Fisher Scientific (United States) and used according to the manufacturer’s recommendations. TaqSE DNA polymerase was from SybEnzyme (Russia), and Q5 high-fidelity DNA polymerase was purchased from New England Biolabs (United States). Plasmid DNA was isolated and purified using a Quantum Prep Plasmid Miniprep Kit (Bio-Rad, United States). The PCR reactions (total volume is 50 μl) were conducted in the following conditions: 200 μM dNTPs, 0.5 μM forward and reverse primers, 0.05 ng/μl *Lysobacter* sp. XL1 genomic DNA (for amplification of a 924-bp DNA fragment containing the 3′ end of *alpB* and downstream region, an 825-bp DNA fragment containing the 5′ end of *alpB* and upstream region and the full-length *alpB* gene with downstream and upstream genome sequences) or 7 ng/μl pBR322 plasmid (for amplification of 1.43 kb Tc^*R*^ cassette), 0.02 U/μl Q5 high-fidelity DNA polymerase (New England Biolabs) in 1 × reaction buffer containing 2 mM MgCl_2_. The thermo cycles were programmed according to the manufacturer’s protocol: initial denaturation at 98°C for 30 s, followed by 30 cycles of 98°C for 10 s, annealing temperature 60°C for 20 s, 72°C for time that was determined by amplicon length (extension times are 30 s per kb), and a final extension at 72°C for 2 min. Amplicons were separated in gels of 0.8% agarose (Merck, Germany) in Tris-acetate-ethylenediaminetetraacetic acid (TAE) buffer containing 0.5 μg/ml ethidium bromide, visualized in a Bio-Print ST4 gel documentation system (Vilber lourmat, France), at 354 nm, and purified by QIAquick spin-column (Qiagen, United States). The concentration of DNA was measured using NanoPhotometer P360 (IMLEN, Germany) and by electrophoretic assay in 0.8% agarose gel.

### Plasmid Constructions

**FIGURE 1:**
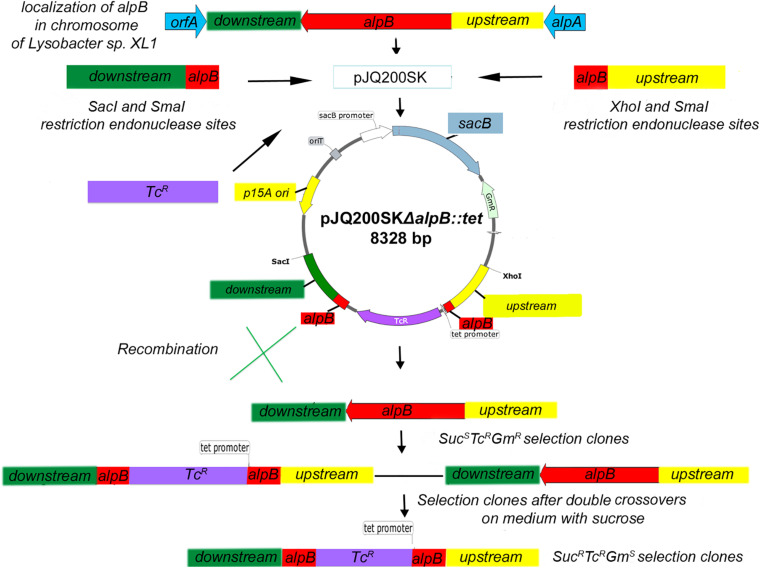
Scheme of inserting a mutation into the *alpB* gene of *Lysobacter* sp. XL1. The pJQ200SKΔ*alpB::tet* recombinant plasmid was introduced into cells of *Lysobacter* sp. XL1 *via* electroporation. Integration of the plasmid into the chromosome was testified by the resistance of transformants to gentamicin (plasmid marker) and tetracycline (cassette marker inserted into the deletion locus) and by the sensitivity to sucrose. As a result of merodiploid resolution, Suc^R^Tc^R^Gm^S^ clones with double crossovers between the mutant and wild-type alleles of the *alpB* gene were selected.

Deletion of *alpB* was done by allelic replacement using a suicide plasmid construct (Figure 1). Briefly, a 924-bp DNA fragment containing the 3′ end of *alpB* and its downstream region was amplified from the genomic DNA of *Lysobacter* sp. XL1 with a pair of primers L5_*Sac*I(for) and L5_*Sma*I(rev). All primers are listed in Supplementary Table 1. The resulting amplicon was ligated into *Sac*I/*Sma*I-digested suicide vector pJQ200SK to form pJQ200SKΔ5′*alpB*. Subsequently, an 825-bp DNA fragment containing the 5′ end of *alpB* and its upstream region was amplified with primers L5_*Sma*I(for) and L5_*Xho*I(rev) and ligated into *Sma*I/*Xho*I-digested pJQ200SKΔ5′*alpB* to give pJQ200SKΔ*alpB*. In pJQ200SKΔ*alpB*, the *alpB* gene carries an 880-bp deletion in its central region. Finally, the deletion allele was marked by the insertion of a 1.43-kb Tc^R^ cassette amplified from pBR322 with primers Tc(for) and Tc(rev), which was phosphorylated with T4 polynucleotide kinase and ligated into *Sma*I-digested pJQ200SKΔ*alpB* pretreated with shrimp alkaline phosphatase to produce the suicide vector plasmid pJQ200SKΔ*alpB*::*tet* (Figure 1 and Supplementary Figure 1A).

To replace the *alpB*::*tet* allele in the deletion mutant with the wild-type *alpB* gene, the full-length *alpB* gene (which is 1,200 bp long) plus its immediate downstream and upstream sequences was amplified using primers L5_*Sac*I(for) and L5_*Xho*I(rev) followed by ligation of the amplicon into the *Sac*I/*Xho*I-digested pJQ200SK to yield pJQ200SKΔ*alpB::alpB* (Supplementary Figure 1B). All cloned DNA fragments were sequenced at the Evrogen JSC (Russia) to confirm the correctness of inserts and the absence of random mutations.

### Bacterial Transformation and Electroporation

*E. coli* cells were transformed by the RbCl method according to Hanahan (1983). *Lysobacter* sp. XL1 electro-competent cells were prepared according to Lin and McBride (1996). Briefly, 20-μl aliquots of *Lysobacter* sp. competent cells in 10% ice-cold glycerol were mixed with 250 ng of pJQ200SKΔ*alpB*::*tet* or pJQ200SKΔ*alpB::alpB* plasmid DNA in 2-mm gapped cuvettes (Bio-Rad, United States). Electroporation was performed using a Gene Pulser apparatus (Bio-Rad, United States) under conditions at 12.5 kV/cm. Immediately after the electric pulse, 1 ml of an LB-M medium was added to the pulsed bacterial cell suspension, which was then incubated for 3 h at 29°C without agitation, and subsequently plated on solid medium containing the appropriate antibiotics.

### Selection of *Lysobacter* sp. XL1Δ*alpB::tet* Deletion Strain and Complementation of Mutation

After electroporation of pJQ200SKΔ*alpB*::*tet* into *Lysobacter* sp. XL1 (or of pJQ200SKΔ*alpB::alpB* into the *Lysobacter* sp. XL1 *alpB*::*tet* mutant), single crossover (SCO) clones with the Suc^S^Tc^R^Gm^R^ phenotype were screened (Figure 1). Double crossover clones (DCOs) with the Suc^R^Tc^R^Gm^S^ phenotype were counter-selected after growing one SCO clone in LB-M broth containing sucrose for 3 h at 29°C, followed by plating and cultivation for 72 h at 29°C on LB-M agar containing Tc. DCOs were confirmed by PCR for the presence of the wild-type or deletion allele using primers in Supplementary Table 1, which amplify a 2.62-kb product from the wild-type allele, or a 3.17-kb product from the mutant allele. The sites of recombination in DCOs were confirmed by sequencing.

### Obtaining Outer Membrane Vesicles

OMVs were isolated by ultracentrifugation from equal volumes of the culture liquids of *Lysobacter* sp. XL1, *Lysobacter* sp. XL1Δ*alpB*::*tet*, and *Lysobacter* sp. XL1Δ*alpB*::*alpB* strains grown in LB-M liquid medium. Briefly, cells from a 0.3-L culture were pelleted by centrifugation at 7,500 × *g* for 20 min at 4°C, and the supernatant was recovered. Vesicles were then pelleted from 300-ml supernatants by centrifugation in an L5-50 ultracentrifuge (Beckman, United States) at 113,000 × *g* for 2 h at 4°C. The OMV pellet was washed once with 50 mM Tris-HCl, pH 8.0, by resuspension and centrifugation at the same speed for 1 h. The washed vesicle pellet was resuspended in 600 μl of 50 mM Tris-HCl, pH 8.0, and stored at –20°C.

### Sodium Dodecyl Sulfate–Polyacrylamide Gel Electrophoresis

For the electrophoretic assay, preparations of wild-type *Lysobacter* sp. XL1 OMVs and *Lysobacter* sp. XL1Δ*alpB::tet* OMVs were sedimented with trichloroacetic acid at a final concentration of 10%. Preparations of OMVs were equal and contained 0.04 mg of total proteins. Protein residues were analyzed by electrophoresis with 0.1% sodium dodecyl sulfate (SDS) in 12.5% polyacrylamide gel (PAG). Electrophoresis in stacking gel was run at 60 V; in separating gel, at 180 V. Protein bands in gels were revealed by staining with a solution of Coomassie Brilliant Blue R-250 (Serva, Germany). As molecular weight markers, we used SM0431 (Thermo Fisher Scientific, United States): β-galactosidase (116 kDa), bovine serum albumin (66.2 kDa), ovalbumin (45 kDa), lactate dehydrogenase (35 kDa), REase Bsp981 (25 kDa), β-lactoglobulin (18.4 kDa), and lysozyme (14.4 kDa).

### Thin-Layer Chromatography

Preparations of wild-type *Lysobacter* sp. XL1 OMVs and *Lysobacter* sp. XL1Δ*alpB::tet* OMVs were aligned by mass of protein (20 μg) for phospholipid extraction. Phospholipids were extracted from both preparations with chloroform–methanol mixture (1:2 v/v) according to the method of Ames (1968). Individual phospholipids were separated by two-dimensional chromatography on silica gel plates 60 F254 (HPTLC; Merck, Germany) using CHCl_3_–CH_3_OH–H_2_O (65:25:4 v/v) mixture as the first-dimension separation system and CHCl_3_–CH_3_OH–CH_3_COOH–H_2_O (40:7.5:6:1.8 v/v) as the second-dimension separation system. Phospholipids were visualized using molybdenum blue solution.

### Quantitation of Outer Membrane Vesicles by Biochemical Analysis

The total protein concentration in OMV preparations was measured by the Lowry method (Lowry et al., 1951) against a standard curve done with bovine serum albumin (Sigma, United States) in the 2–20 μg/ml range.

The concentration of 2-Keto-3-deoxyoctanate (Kdo, a core LPS component) was determined by the reaction with thiobarbituric acid, exactly as described by Karkhanis et al. (1978). The standard curve was done with an aqueous solution of Kdo (Sigma, United States) in the 1.85–29.60 μg/ml range.

### Measurement of Lytic Activity

The total bacteriolytic activities of culture supernatants were determined by turbidimetry as previously reported (Vasilyeva et al., 2014). Briefly, liquid cultures of *Lysobacter* strains in LB-M were centrifuged at 7,500 × *g* for 20 min at 4°C to pellet bacterial cells. Then, 25 μl of the clear supernatant were added to 975 μl of heat-killed cells of *S. aureus* 209P suspended to an OD_540_ = 0.5 in 10 mM Tris-HCl, pH 8.0, and incubated at 37°C for 5 min. The reaction was arrested by placing test tubes on ice, and the OD_540_ of the suspension was measured in a Beckman DU 730 (United States) spectrophotometer. A decrease in 0.01 optical units/min (at 37°C) was taken as a lytic unit (LU). Therefore, LUs were calculated by the following formula: {[0.5 (initial OD_540_ of suspension) – final OD_540_] × 1,000 μl (total reaction volume)}/[5 min (time of reaction) × 25 μl (volume of sample) × 0.01 (correction coefficient for OD reduction per minute)].

The lytic action of OMVs against live Gram-positive bacteria was determined by the spot assay with *S. aureus* 209P, *M. roseus* B-1236, *M. luteus* B-1813, and *B. cereus* B-454 (Vasilyeva et al., 2014). Three independent experiments were carried out. Vesicle preparations (as 12-μl aliquots) were applied on a pre-grown lawn of the target bacterium on an agar plate after the concentration of OMVs had been adjusted to have identical amounts (0.09 μg) of Kdo. Applied OMV samples were allowed to absorb into the lawn for 30 min, and plates were then incubated at 29°C overnight (16 h). The lytic action of the preparations was then assessed by simple observation where the presence of a clear lysis zone indicated strong activity, a hazy lysis zone indicated weak activity, and the absence of a lysis zone indicated no activity.

### Transmission Electron Microscopy

Samples of OMVs (obtained from two independent isolation runs) of *Lysobacter* sp. XL1, *Lysobacter* sp. XL1Δ*alpB*::*tet*, and *Lysobacter* sp. XL1Δ*alpB*::*alpB* were diluted fivefold in 50 mM Tris-HCl, pH 8.0, and 10 μl were placed on top of a formvar-coated copper grid. The applied sample was allowed to adsorb for 2 min, and sample excess was then removed using filter paper. After air-drying, the samples were stained with 10 μl of 0.3% aqueous solution of uranyl acetate (pH 4.0), placed on the grids, and immediately removed using filter paper. Negatively stained preparations were examined with a JEM-1400 transmission electron microscope (JEOL, Japan) at an accelerating voltage of 80 kV, and random images of representative fields of observation were captured with an 11-Megapixel TEM Camera MORADA G2 (EMSIS GmbH, Germany). All micrographs were done with 25,000 magnification. The six micrographs were obtained from each experiment. All vesicles of wild-type *Lysobacter* sp. XL1, *Lysobacter* sp. XL1Δ*alpB::tet*, and *Lysobacter* sp. XL1Δ*alpB::alpB* in all obtained micrographs were studied. Ultrastructural observations, including various degrees of density (full and empty vesicles), and measurements were done on the acquired images. The ratio of full/empty vesicles was estimated by visual analysis of micrographs: full OMVs have compact packaging or local crystal packing, and in the case of the empty OMVs, it was possible to distinguish the backing film of the grid.

### Statistical Analysis

All experiments were in triplicates. Statistical analysis was done using GraphPad Prism (version 9.1.0; GraphPad Software, San Diego, CA). The analytical results were compared by Student’s *t*-test. Data are presented as the mean ± standard deviation. Statistical significance was considered at *p* < 0.05.

## Results

### *alpB* Is Not an Essential Gene in *Lysobacter* sp. XL1

**FIGURE 2:**
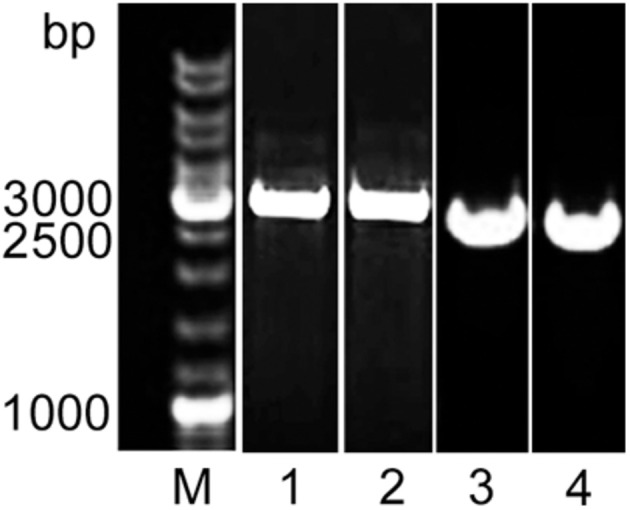
Molecular evidence of the deletion of *alpB* in *Lysobacter* XL1. Agarose gel stained with ethidium bromide after the electrophoresis of PCR amplicons produced with primers L5_*Sac*I(for) and L5_*Xho*I(rev). The PCR amplicon for the mutant allele is 3,177 bp long (including *alpB* flanking sequences + the TcR cassette), whereas the wild-type allele produces a 2,623-bp product (including the full-length *alpB* gene + adjacent regions). M, SM0331 GeneRuler DNA Ladder Mix (Thermo Fisher Scientific, United States) (lane taken from Supplementary Figure 3); **(1)** *Lysobacter* sp. XL1Δ*alpB::tet* clone (from Supplementary Figure 2); **(2)** pJQ200SKΔ*alpB::tet* plasmid (from Supplementary Figure 2); **(3)** *Lysobacter* sp. XL1Δ*alpB::alpB* clone (from Supplementary Figure 3); and **(4)** *Lysobacter* sp. XL1 clone (from Supplementary Figure 3).

The deletion of *alpB* in *Lysobacter* sp. XL1Δ*alpB*::*tet* was genetically confirmed by the production of a 3,177-bp PCR amplicon (Figure 2) that was subsequently sequenced to show that the Tc^R^ cassette had indeed replaced, as expected, the region between 117 and 997 bp of the *alpB* gene. *Lysobacter* sp. XL1Δ*alpB::tet* grew well in LB-M (Table 2), indicating that *alpB* is not an essential gene and does not affect the gross overall physiology of the bacterium. The mutation was confirmed biochemically and functionally by the reduction in bacteriolytic activity of the mutant strain (Table 2 and Supplementary Figure 4). Since the total lytic activity of *Lysobacter* sp. XL1 is attributed to several secreted bacteriolytic enzymes (reviewed by Vasilyeva et al., 2013), it seems reasonable to surmise that the observed difference in lytic activities between *Lysobacter* sp. XL1 and *Lysobacter* sp. XL1Δ*alpB::tet* corresponds to the activity contributed by protein L5 (AlpB).

**TABLE 2:** Effect of *alpB* gene knockout on *Lysobacter* sp. XL1 growth and bacterial activity.

Strains	OD_540_	LU/ml
*Lysobacter* sp. XL1Δ*alpB::tet*^a^	4.65 ± 0.09	115.68 ± 18.82
Wild-type *Lysobacter* sp. XL1^b^	3.81 ± 0.81^ns^	149.00 ± 14.85***
*Lysobacter* sp. XL1Δ*alpB::alpB*^c^	4.59 ± 0.28^ns^	140.00 ± 0.92*

Results are shown as mean ± *standard deviation. The mean values were obtained in three independent experiments, each with two technical replicates (n* = *6). ^*ns*^ Not statistically significant* = *p* > *0.05 when comparing the means of the two groups (^*a*^ and ^*b*^—OD_540_; ^*a*^ and ^*c*^—OD_540_) by the Student’s t-*test. *** p < *0.001 when comparing the means of the two groups (^*a*^ and ^*b*–^LU/ml) by the Student’s t-*test. *p < *0.05 when comparing the means of the two groups (^*a*^ and ^*c*^—LU/ml) by the Student’s t-*test.

### Effect of *alpB* Gene Knockout on Vesicle Formation of *Lysobacter* sp. XL1

Vesicle preparations obtained from equal volumes of the culture liquids of the wild-type, complemented strain, and mutant strains were analyzed by TEM and characterized biochemically.

**FIGURE 3:**
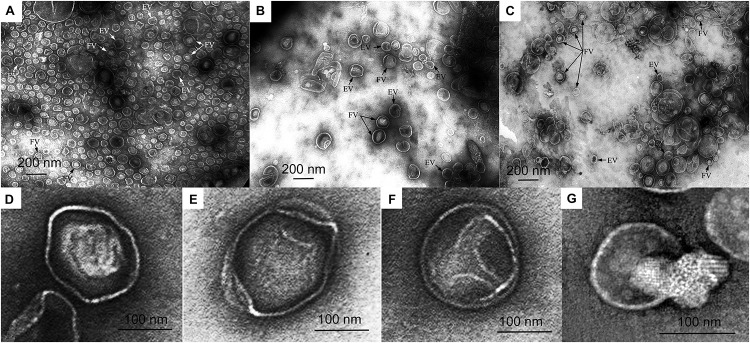
Electron microscopy of vesicle preparations. **(A)** Wild-type *Lysobacter* sp. XL1 vesicles. **(B)** *Lysobacter* sp. XL1Δ*alpB::tet* mutant strain vesicles. **(C)** *Lysobacter* sp. XL1Δ*alpB::alpB* complemented strain vesicles. **(D)** Representative outer membrane vesicle (OMV) with a clear-cut dense polygonal core. **(E)** Representative OMV with diffusely distributed low-density contents. **(F)** Representative OMV with small contents in the form of a small loose granule at the periphery inside the vesicle. **(G)** Representative empty (or broken) vesicle that seems to have spilled its contents. Vesicles from **(D–G)** are from the mutant strain. EV, empty vesicle; FV, full vesicle.

In the preparation of mutant strain vesicles, the predominant species are larger vesicles 100–150 nm (47%) and 150–190 nm (30%) in diameter (Figure 3B and Supplementary Figure 5A). Vesicles 50–100 nm in diameter are few (14%). Vesicles in the preparation produced from the wild-type culture liquid are predominantly 50–100 nm (73%) and 100–150 nm (24%) in diameter (Figure 3A and Supplementary Figure 5B). Large vesicles 150–190 nm in diameter are 3% of the total number of vesicles occurring in all fields of vision. Vesicles in the preparation produced from the complemented strain are two groups as wild-type: 100–150 nm (46%) and 50–100 nm (33%) (Figure 3C and Supplementary Figure 5C). Vesicles 150–190 nm and 190–250 nm in diameter are few (17 and 4%, respectively).

The main distinction of the preparations is the various degrees of density (full and empty vesicles). The micrographs in Figures 3D–G show OMVs with representative ultrastructures observed in all preparations. Vesicles with clear-cut dense cores of polygonal shapes occur (Figure 3D). Such structures bear resemblance to crystals and may form as a result of a dense packing of their contents. Besides these, vesicles with diffusely distributed contents of low densities (Figure 3E) and with small contents in the form of a small loose granule at the periphery inside the vesicle (Figure 3F) occur, as well as broken vesicles with their contents released outside (Figure 3G). Although all these types of vesicles were observed in all preparations, they were present in very different proportions. TEM obtained that the preparations of wild-type OMVs and complemented strain OMVs contain more full vesicles than that of mutant strain vesicles. Thus, the ratio of full/empty vesicles in the wild-type strain and complemented strain is 2, whereas in the mutant strain, it is 0.7. This analysis considered only intact vesicles.

The main components of vesicles are proteins, lipids, and LPS. By the content of protein and Kdo (the essential constituent of LPS) in the preparations, we can assess the quantity of formed vesicles. The general analysis of protein revealed that its content in the vesicle preparation of the mutant strain is 2.4 times and 1.6 times lower than that in the vesicle preparation of the wild-type strain and complemented strain, respectively (Table 3 and Supplementary Figure 6). The content of Kdo in the vesicle preparation of the mutant strain is 2.1 times and 1.6 times lower than in the vesicle preparation of the wild-type strain and complemented strain, respectively (Table 3 and Supplementary Figure 7).

**TABLE 3:** Content of protein and Kdo in OMV preparations.

OMVs	Total protein content, mg/ml	Concentration of Kdo (μg/ml)
*Lysobacter* sp. XL1Δ*alpB::tet*^a^	0.39 ± 0.07	8.25 ± 2.49
Wild-type *Lysobacter* sp. XL1^b^	0.95 ± 0.04***	17.35 ± 3.73***
*Lysobacter* sp. XL1Δ*alpB::alpB*^c^	0.62 ± 0.07***	12.84 ± 2.68*

Results are shown as mean ± *standard deviation. The mean values were obtained in three independent experiments, each with two technical replicates (n* = 6). *** p < *0.001 when comparing the means of the two groups (^*a*^ and ^*b*^—total protein content and Kdo concentration; ^*a*^ and ^*c*^—total protein content) by the Student’s t-*test. *p < *0.05 when comparing the means of the two groups (^*a*^ and ^*c*^—Kdo concentration) by the Student’s t-*test.

The general electrophoretic pattern for the distribution of major proteins in the vesicle preparation of the mutant strain did not differ from that in the wild-type strain. The phospholipid composition of mutant strain vesicles did not change either (Supplementary Figure 8). The main phospholipid of vesicles of the wild-type and mutant strains is cardiolipin.

In summary, the knockout of the *alpB* gene led to a decrease of the quantity of formed vesicles and to a change in the degree to which they are filled.

The obtained results confirm the influence of protein L5 on vesicle biogenesis of *Lysobacter* sp. XL1.

### Effect of *alpB* Gene Knockout on the Lytic Properties of Vesicles

**FIGURE 4:**
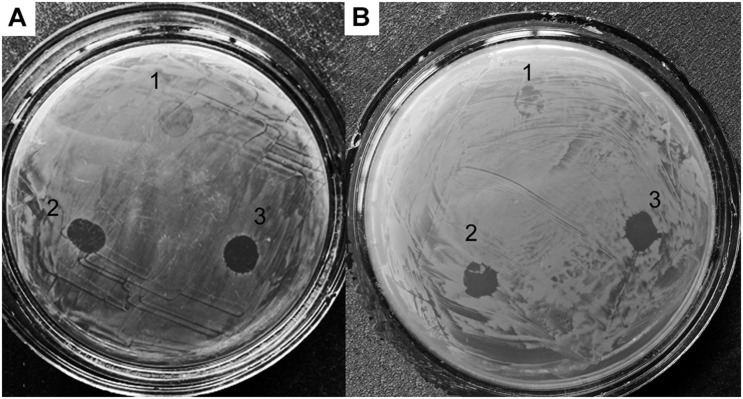
Lytic activities of *Lysobacter* sp. XL1Δ*alpB*::*tet* **(1)**, *Lysobacter* sp. XL1Δ*alpB::alpB* **(2)**, and wild-type *Lysobacter* sp. XL1 **(3)** vesicle preparations with respect to live test cultures. **(A)** *S. aureus* 209P. **(B)** *M. luteus* B-1813.

To study the effect of *alpB* gene knockout on the lytic activity of vesicles, as test cultures, we used living cells of several bacteria (Table 4 and Figure 4).

**TABLE 4:** Lytic action of vesicles.

Bacteria	OMV preparations		
	Wild-type *Lysobacter* sp. XL1	*Lysobacter* sp. XL1Δ*alpB::tet*	*Lysobacter* sp. XL1Δ*alpB::alpB*
*S. aureus* 209P	++	±	++
*M. luteus* B-1813	++	±	++
*M. roseus* B-1236	++	±	++
*B. cereus* B-454	++	±	++

*++, strong lytic effect;±, weak lytic effect.*

As seen in the figure and table, vesicles of the mutant strain practically lost their ability to lyse living target cells, whereas vesicles of the wild-type and complemented strains possess a strong lytic action. Thus, knockout of the *alpB* gene was accompanied by a significant decrease of vesicles’ lytic properties, in addition to the reduction of the antimicrobial potential of *Lysobacter* sp. XL as a whole.

## Discussion

The results presented in this work continue the investigation of the influence of bacteriolytic enzyme L5 on the vesicle biogenesis in the Gram-negative bacterium *Lysobacter* sp. XL1.

Based on our earlier work, we proposed that protein L5 could be involved in the biogenesis of the subpopulation of vesicles by means of which it was released into the extracellular milieu and proposed a model of the process. Immediately, a question arose how enzyme L5 could initiate the formation of vesicles. One of the existing models of vesicle biogenesis in Gram-negative bacteria assumes a pressure of cell debris (misfolded proteins, peptidoglycan, and LPS fragments) on the inner leaflet of the OM, which provokes vesicle formation (Zhou et al., 1998; Hayashi et al., 2002; Schwechheimer et al., 2014). At some point, we assumed that vesicle biogenesis in *Lysobacter* sp. XL1 could occur according to that model. However, on the premise that the OM is a dynamic and well-regulated cell structure of complex organization, it is impossible to imagine that a pressure caused by accumulation of protein at the inner leaflet of the OM could evoke vesicle formation. In our view, this single condition is insufficient.

Further investigation of the biogenesis revealed that the main phospholipid of *Lysobacter* sp. XL1 vesicles was cardiolipin (Kudryakova et al., 2017). That meant that vesicles formed from OM loci enriched with this phospholipid. The hydrophilic head of cardiolipin carries two negative charges, and, due to their intermolecular repulsion, the rigidity of the OM can be disturbed in loci enriched in this phospholipid, which leads to the emergence of a destabilization locus. According to two more known models, vesicles form from certain loci of OM destabilization, which can emerge due to a decreased lipoprotein content (Hoekstra et al., 1976; Deatherage et al., 2009; Schwechheimer et al., 2014) or an increased content of curvature-inducing molecules (B-type LPS, signaling molecule PQS) (Kadurugamuwa and Beveridge, 1995; Mashburn-Warren and Whiteley, 2006; Schertzer and Whiteley, 2012). We note here once again that the cell envelope has a complex organization, and the occurrence of destabilization loci leading to spontaneous evagination and release of OM fragments is not probable. Thus, it is also hard to consider that destabilization loci in the OM are the sole condition for vesicle formation.

**FIGURE 5:**
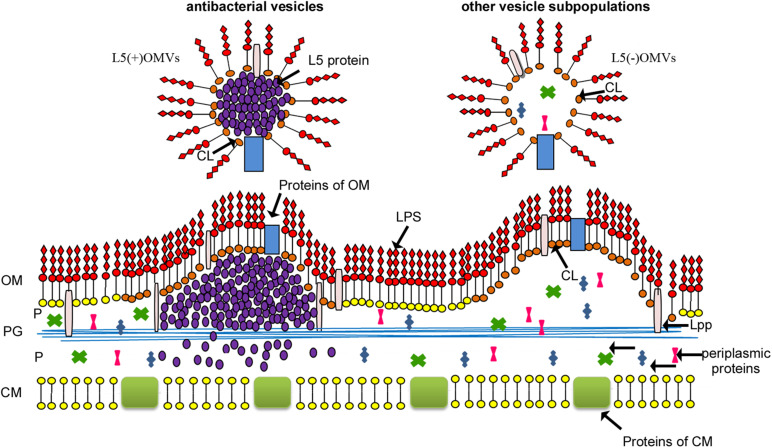
Model of *Lysobacter* sp. XL1 vesicle biogenesis (Kudryakova et al., 2015) with modification. *Lysobacter* sp. XL1 forms several subpopulations of vesicles from OM loci enriched with CL. The hydrophilic head of cardiolipin carries two negative charges, and, due to their intermolecular repulsion, the rigidity of the OM can be disturbed in loci enriched with this phospholipid, which leads to the initiation of vesicle formation (Kudryakova et al., 2017). One of the vesicle subpopulations contains bacteriolytic enzyme L5. In the process of its topogenesis, this protein accumulates in the periplasm at the inner leaflet of the OM (Kudryakova et al., 2015) in the form of amyloid-like structures (Kudryakova et al., 2018). This may lead to a pressure on the cardiolipin-enriched inner leaflet of the OM (the destabilization locus) and contribute to the formation of a subpopulation of antibacterial vesicles containing protein L5. This vesicle subpopulation is a “bacterial bomb” for competitive bacteria and is involved in invasion expansion of ecological niches for *Lysobacter* sp. XL1. L5(+)OMVs, antibacterial subpopulation of vesicles containing protein L5; L5(–)OMVs, other subpopulations of vesicles; LPS, lipopolysaccharide; OM, outer membrane; P, periplasm; CM, cytoplasmic membrane; Lpp, lipoprotein; PG, peptidoglycan; CL, cardiolipin.

Instead, we showed that *Lysobacter* sp. XL1 vesicles formed from cardiolipin-enriched OM loci, and a particular subpopulation contained bacteriolytic protein L5 (Figure 5). Based on these results, we suggested that vesicle formation was due to a set of several factors. An obligatory factor is the occurrence of an OM destabilization locus, and a factor initiating vesicle formation is a component of the inner contents; in the case of *Lysobacter* sp. XL1, it is bacteriolytic enzyme L5. We should single out here one more vesicle biogenesis model based on specific sorting of vesicles’ inner components, proposed by American scientists Amanda Horstman and Meta Kuehn (Horstman and Kuehn, 2000). Their idea is intensively developed now (Haurat et al., 2011, 2015; Evans et al., 2012; Elhenawy et al., 2014; Schwechheimer and Kuehn, 2015). In our opinion, at present, this model can be considered to be the most significant for understanding vesicle biogenesis. It is proposed to be supplemented by the idea that vesicles form in the OM destabilization locus, and additionally, a vesicle-formation initiator is a sorted vesicle component. Other results can be taken to be in favor of this concept. Haurat et al. (2011) present possible models of selective protein sorting due to the occurrence of domains, which can interact with B-type LPS (from which destabilization loci form). The work considers the selective sorting of gingipain into *Porphyromonas gingivalis* vesicles. Besides, the same authors assume the occurrence of a sorting factor, which binds protein directed into vesicles to B-type LPS (Haurat et al., 2015). In this model, they draw an analogy with the role of galectin in protein sorting in the formation of eukaryotic exosomes. Those authors were the first to show a relation of the prospective vesicle component (the sorting component) with B-type LPS, which leads to OM destabilization. Our model assumes that alkaline protein L5 possesses some affinity to the acid cardiolipin. Notably, studies of the structural and functional features of this enzyme have revealed the formation of amyloid-like structures with increased concentration. It has been found that, in vesicles, this enzyme is namely in its amyloid-like form (Kudryakova et al., 2018). This property can be assumed to be important for initiating the formation of vesicle in the destabilization locus. However, proof of this requires integrated molecular, genetic, and structural studies, which will enable understanding the accurate mechanism of the process.

Additional confirmation that protein L5 possesses an ability to initiate vesicle formation is the result of the expression of enzyme L5 in the recombinant strain *P. fluorescens* Q2-87/B (Kudryakova et al., 2017). As the result of expression of this protein gene, the recombinant strain formed a larger number of vesicles, which produced enzyme L5 and acquired lytic properties.

To confirm the role of protein L5 in *Lysobacter* sp. XL1 vesicle biogenesis, we lacked genetic studies, in particular, of the knockout of a corresponding gene. The generation of gene knockouts in *Lysobacter* spp. constitutes a very recent development, as the availability of molecular genetic tools for this bacterium has evolved slowly. Only a few investigations to date are known to involve the deletion of *Lysobacter* genes, like the chitinase gene and the genes responsible for the biosynthesis of antibiotics and for regulation of this process in *L. enzymogenes* OH11 (Qian et al., 2012; Xu et al., 2015; Wang et al., 2016, 2017). Therefore, the successful deletion of *alpB* represents an important contribution to this field of genetic investigation.

In this work, we present the results of research into the effect of bacteriolytic enzyme L5 gene knockout on vesicle formation in *Lysobacter* sp. XL1. As a result of this gene knockout, the total bacteriolytic activity of the culture liquid in the mutant strain decreased. The following results became crucial for our hypothesis: the mutant strain formed fewer vesicles, the degree of their filling decreased, the lytic properties of vesicles were lost practically completely. The latter result indicates that, in the mutant strain, there is no subpopulation of vesicles containing enzyme L5. However, other subpopulations of vesicles continued to form.

Thus, we conclude that bacteriolytic enzyme L5 influences the formation of vesicle subpopulations that contain it. The molecular mechanisms of this effect are yet to be established by future research.

## Data Availability Statement

The raw data supporting the conclusions of this article will be made available by the authors without undue reservation.

## Author Contributions

IK, AA, and NL contributed to planning the experiments. IK, AA, and TI contributed to the experimental work. EL contributed to the purification of vesicles. NS contributed to the electron microscopy. NL contributed to the project administration. IK contributed to the funds acquisition. IK and NL contributed to the writing the manuscript. All authors contributed to the article and approved the submitted version.

## Conflict of Interest

The authors declare that the research was conducted in the absence of any commercial or financial relationships that could be construed as a potential conflict of interest.

## Publisher’s Note

All claims expressed in this article are solely those of the authors and do not necessarily represent those of their affiliated organizations, or those of the publisher, the editors and the reviewers. Any product that may be evaluated in this article, or claim that may be made by its manufacturer, is not guaranteed or endorsed by the publisher.
