# Supplementary material for: Deletion of alpB Gene Influences Outer Membrane Vesicles Biogenesis of Lysobacter sp. XL1
Source: Front Microbiol. 2021 Aug 16;12:715802. doi: 10.3389/fmicb.2021.715802 (PMC8415630; doi:10.3389/fmicb.2021.715802)
Supplement: Supplementary file 1 [file Data_Sheet_1.PDF]

Supplementary Material

Supplementary Table 1. Oligonucleotides used in this work for cloning and sequencing.

Oligonucleotides used for cloning		
**Primers**	**Sequence**	**Purpose**
L5_SacI(for)<br>L5_SmaI(rev)	AATGAGCTCCTGGCCACGACTCTC<br>TGACCCGGGCCAACAATTGC	To amplify a 924 bp fragment containing the 3′ end of *alpB* and its downstream region from *Lysobacter* sp. XL1 DNA
L5_SmaI(for)<br>L5_XhoI(rev)	AACCCGGGCGAGCTTGAGAGTCG<br>TTCTCGAGTCGAACGGCAACAACTG	To amplify a 825 bp fragment containing the 5′ end of *alpB* and its upstream region from *Lysobacter* sp. XL1 DNA
Tc(for)<br>Tc(rev)	GAATTCTCATGTTTGACAGCTTATCATCGA<br>CCCGAGATGCGCCG	To amplify a 1433 bp Tc cassette from plasmid pBR322
L5_SacI(for)<br>L5_XhoI(rev)	AATGAGCTCCTGGCCACGACTCTC<br>TTCTCGAGTCGAACGGCAACAACTG	To amplify a 2 623 bp *alpB* and flanking gene *alpB* sequences from genomic *Lysobacter* sp. XL1 DNA
**Oligonucleotides used for sequencing**		
L5_SacI(for)	AATGAGCTCCTGGCCACGACTCTC	To confirm the correctness of the cloned fragments and the absence of random mutations
L5_SmaI(rev)	TGACCCGGG CCAACAATTGC	
L5_SmaI(for)	AACCCGGGCGAGCTTGAGAGTCG	
L5_XhoI(rev)	TTCTCGAGTCGAACGGCAACAACTG	
L5_seq(for)	ATGTCCGTATCAAAGTCGAATCTGC	
L5_seq(rev)	TCAGCTCGTGACCAGGG	
Mut(for)	GCATGCGTCTCGACCAAAG	
Mut(rev)	GGCCTGTGTCGCGATTGAT	

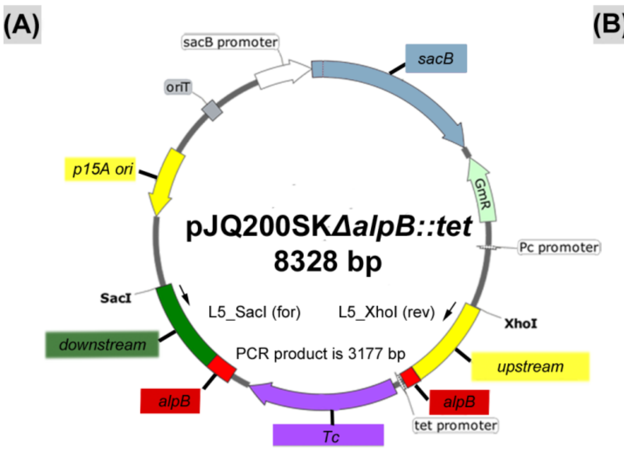

Supplementary Figure 1. Scheme of constructed plasmids. **(A)** Plasmid pJQ200SK*ΔalpB::tet* produced by cloning a 924 bp DNA fragment containing the 3′ end of *alpB* and its downstream region into the SacI/SmaI restriction sites, an 825 bp DNA fragment containing the 5′ end of *alpB* and its upstream region into the SmaI/XhoI restriction sites, marking of the deletion by insertion of 1.43 kb Tc^R cassette into the SmaI restriction site. **(B)** Plasmid pJQ200SK*ΔalpB::alpB* produced by cloning the full-length *alpB* gene with downstream and upstream genome sequences into the SacI/XhoI restriction sites.

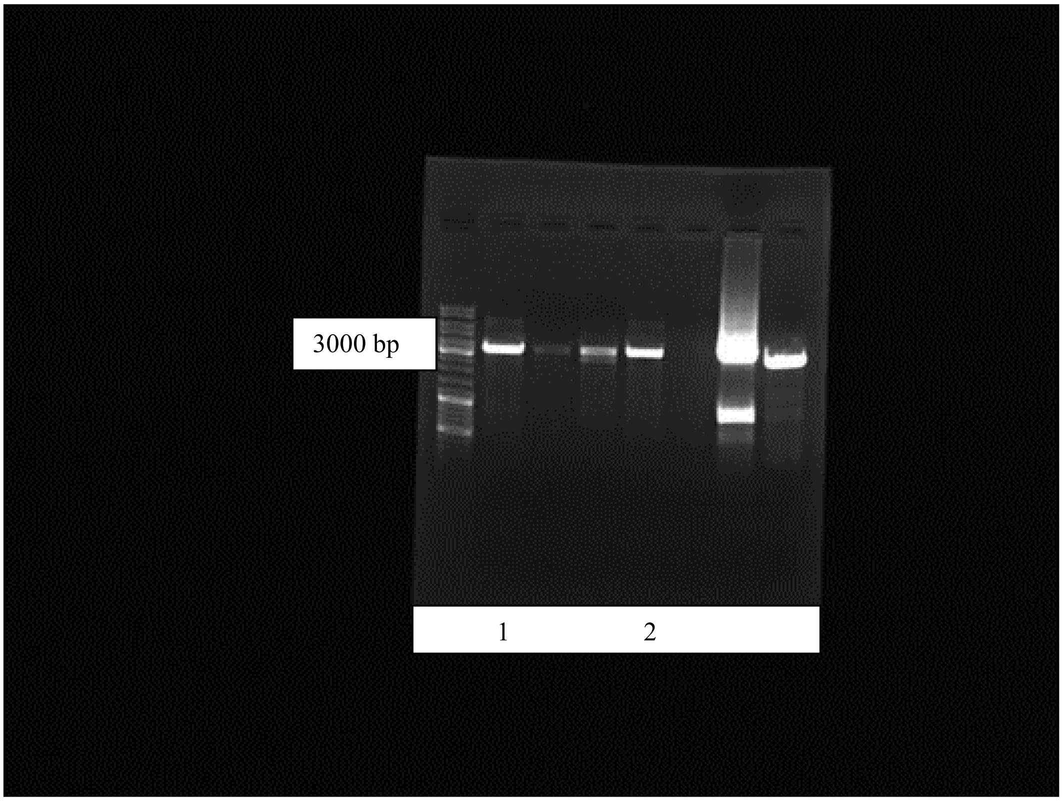

Supplementary Figure 2. Original gel images for Fig. 2: lanes 1, 2 correspond to lanes 1, 2 of Figure 2.

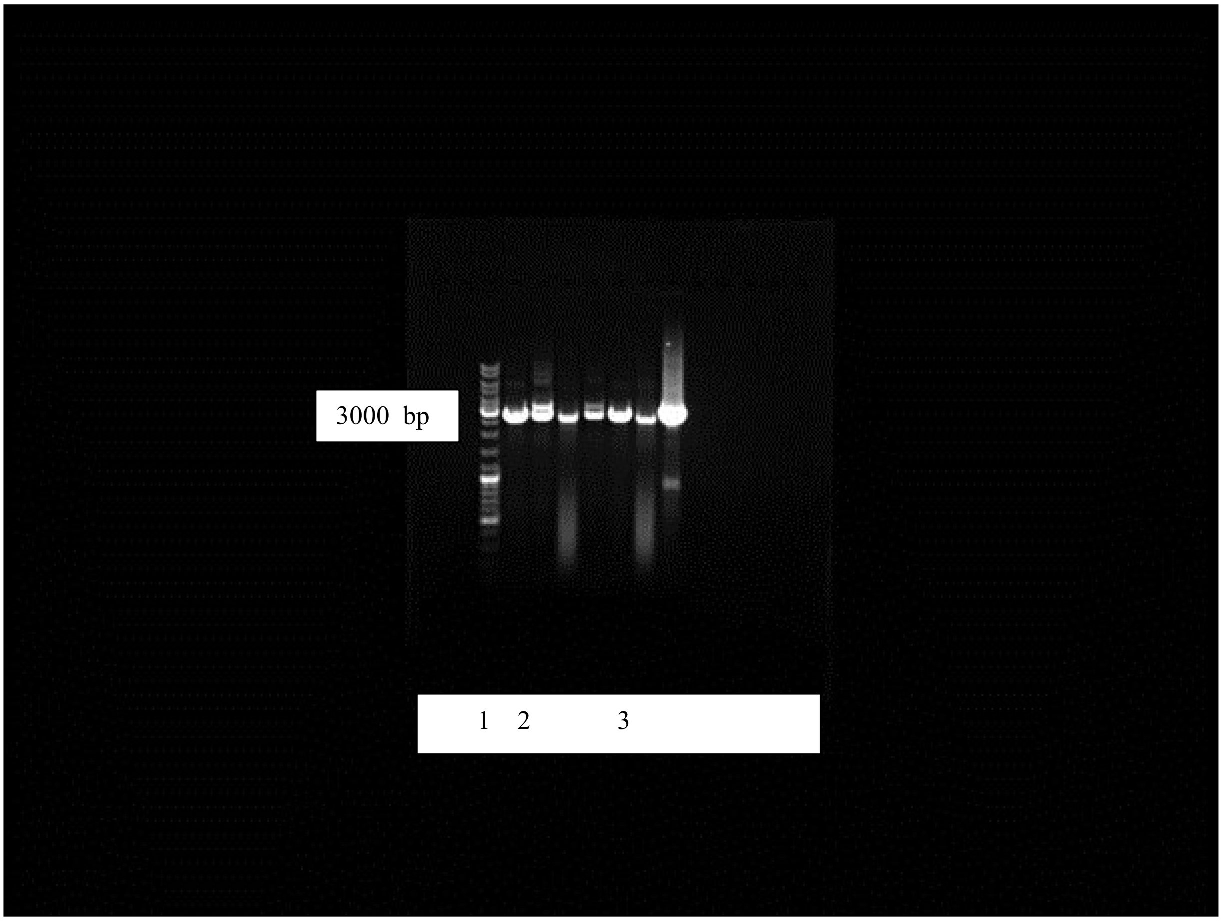

Supplementary Figure 3. Original gel images for Fig. 2: lanes 1, 2, 3 correspond to lanes M, 3, 4 of Figure 2.

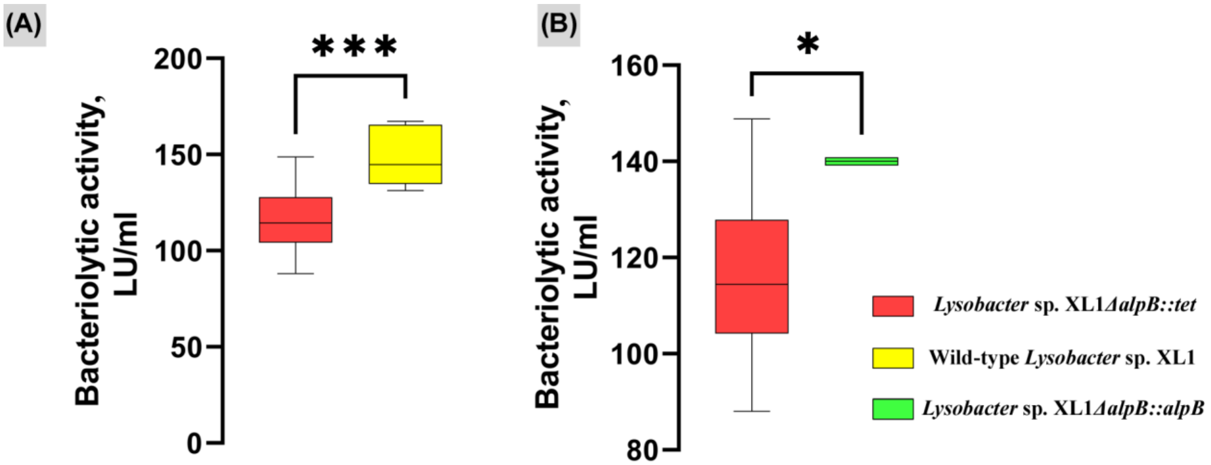

Supplementary Figure 4. Box plot graph of the total bacteriolytic activity (indicated as lytic units per ml) of *Lysobacter* sp. XL1*ΔalpB::tet* and wild-type *Lysobacter* sp. XL1 **(A)**; *Lysobacter* sp. XL1*ΔalpB::tet* and *Lysobacter* sp. XL1*ΔalpB::alpB* **(B).** Statistical significance is indicated by asterisks as follows: ***$P < 0.001$. * $P < 0.05$.

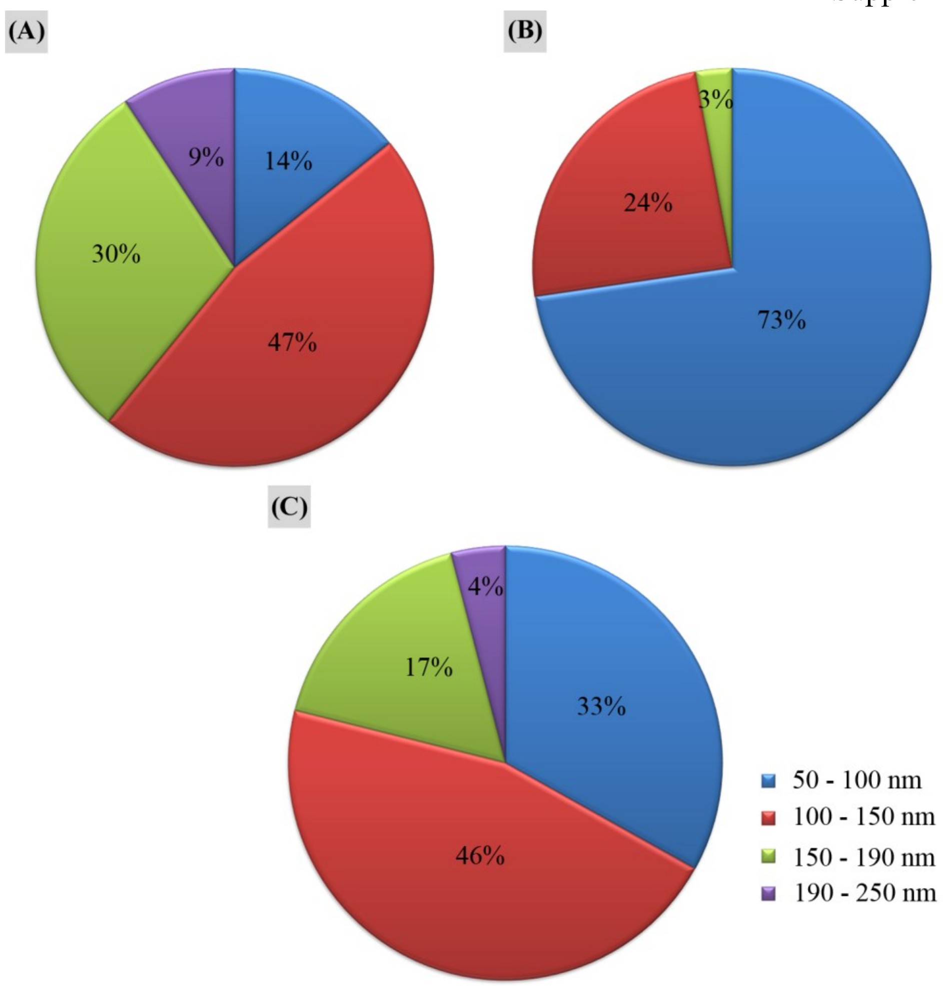

Supplementary Figure 5. Pie chart of the distribution of vesicles by size. **(A)** *Lysobacter* sp. XL1*ΔalpB::tet* mutant strain vesicles. **(B)** Wild-type *Lysobacter* sp. XL1 vesicles. **(C)** *Lysobacter* sp. XL1*ΔalpB::alpB* complemented strain.

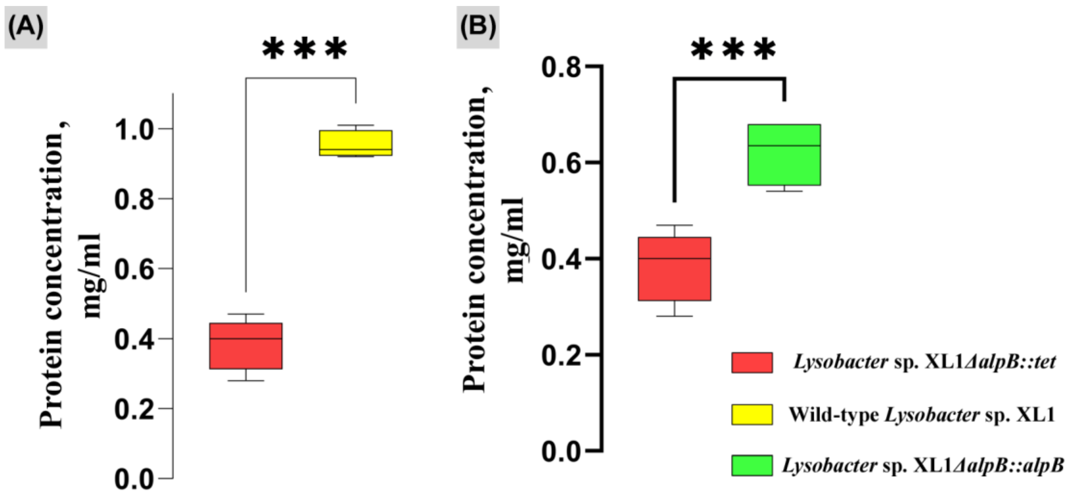

Supplementary Figure 6. Box plot distribution of the protein concentration of *Lysobacter* sp. XL1*ΔalpB::tet* and wild-type *Lysobacter* sp. XL1 **(A)**; *Lysobacter* sp. XL1*ΔalpB::tet* and *Lysobacter* sp. XL1*ΔalpB::alpB* **(B).** Statistical significance is indicated by asterisks as follows: ***$P < 0.001$.

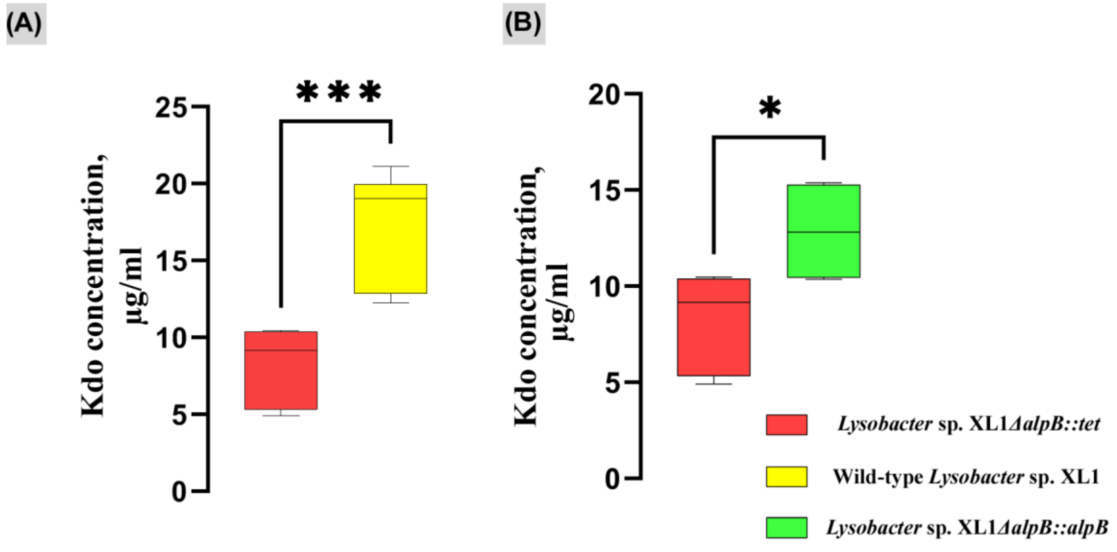

Supplementary Figure 7. Box plot distribution of the Kdo concentration of *Lysobacter* sp. XL1*ΔalpB::tet* and wild-type *Lysobacter* sp. XL1 **(A)**; *Lysobacter* sp. XL1*ΔalpB::tet* and *Lysobacter* sp. XL1*ΔalpB::alpB* **(B).** Statistical significance is indicated by asterisks as follows: ***$P < 0.001$. * $P < 0.05$.

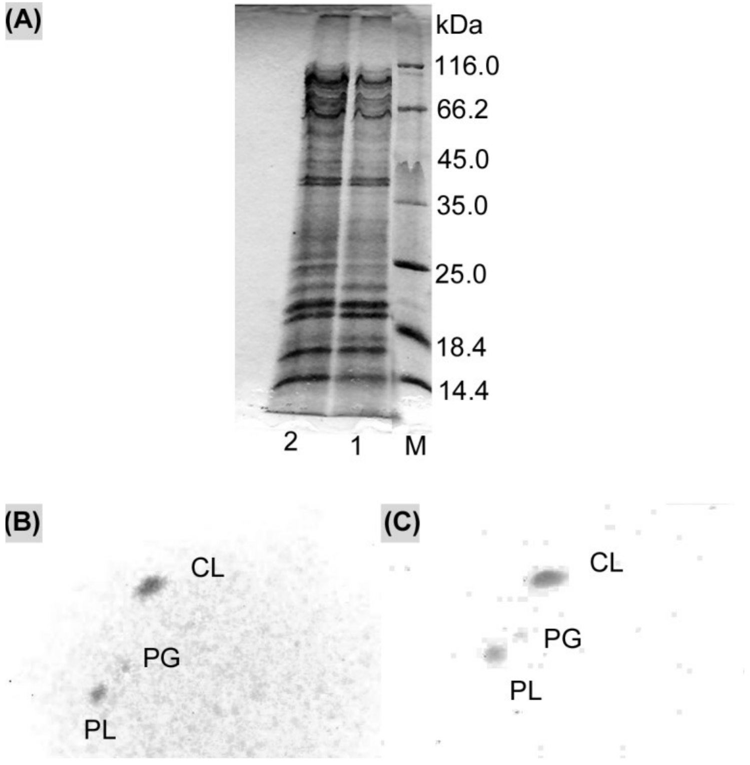

Supplementary Figure 8. Comparison of wild-type *Lysobacter* sp. XL1 vesicles and *Lysobacter* sp. XL1*ΔalpB::tet* mutant strain vesicles. **(A)** SDS-PAGE. A comparative electropherogram of protein content in OMVs preparation of wild-type *Lysobacter* sp. XL1 (1) and *Lysobacter* sp. XL1*ΔalpB::tet* (2). The samples contained 0.04 mg protein.

TLC of phospholipids of *Lysobacter* sp. XL1*ΔalpB::tet* OMVs **(B)** and wild-type *Lysobacter* sp. XL1 OMVs **(C).** CL, cardiolipin; PG, phosphatidylglycerol; PL, unidentified phospholipids. Preparations of OMVs were aligned by mass of protein (20 µg) for extraction of phospholipids.
